# Participation of miR165a in the Phytochrome Signal Transduction in Maize (*Zea mays* L.) Leaves under Changing Light Conditions

**DOI:** 10.3390/ijms25115733

**Published:** 2024-05-24

**Authors:** Dmitry N. Fedorin, Alexander T. Eprintsev, Victoria O. Chuykova, Abir U. Igamberdiev

**Affiliations:** 1 Department of Biochemistry and Cell Physiology, Voronezh State University, 394018 Voronezh, Russia; rybolov@mail.ru (D.N.F.); bc366@bio.vsu.ru (A.T.E.); v.chuykova2020@mail.ru (V.O.C.); 2 Department of Biology, Memorial University of Newfoundland, St. John’s, NL A1C 5S7, Canada

**Keywords:** Arabidopsis, microRNA, phytochrome, signal transduction, extracellular vesicles, *Zea mays* L.

## Abstract

The involvement of the microRNA miR165a in the light-dependent mechanisms of regulation of target genes in maize (*Zea mays*) has been studied. The light-induced change in the content of free miR165a was associated with its binding by the AGO10 protein and not with a change in the rate of its synthesis from the precursor. The use of knockout Arabidopsis plants for the phytochrome A and B genes demonstrated that the presence of an active form of phytochrome B causes an increase in the level of the RNA-induced silencing miR165a complex, which triggers the degradation of target mRNAs. The two fractions of vesicles from maize leaves, P40 and P100 that bind miR165a, were isolated by ultracentrifugation. The P40 fraction consisted of larger vesicles of the size >0.170 µm, while the P100 fraction vesicles were <0.147 µm. Based on the quantitative PCR data, the predominant location of miR165a on the surface of extracellular vesicles of both fractions was established. The formation of the active form of phytochrome upon the irradiation of maize plants with red light led to a redistribution of miR165a, resulting in an increase in its proportion inside P40 vesicles and a decrease in P100 vesicles.

## 1. Introduction

MicroRNAs (miRNAs) are single-stranded RNAs, 20–22 nucleotides in length, that regulate gene expression in various ways. Currently, several thousand miRNAs have been described, including light-dependent ones, such as miR165a [1]. One of the main functions of miRNAs is attaching as part of the RNA-induced silencing complex (RISC) to the 3′-untranslated end of mRNA, during which the latter is degraded or the translation process is blocked. Non-coding RNA molecules, through the mechanism of epigenetics, influence gene expression without altering nucleotide sequences [2]. MiRNAs are involved in the light regulation of plant metabolism. Studies of the mechanisms of light signal perception and transmission in plants have demonstrated the presence of a coordinated signaling network that affects many types of regulatory systems. Different types of photoreceptors sense a wide range of visible light wavelengths. In addition to photoreceptors initiating the signal transduction, the molecules participating in the transmission of light signals include transcription factors (TFs) such as the phytochrome interacting factor (PIF), the bZIP-G-box binding factor (GBF), and other regulatory compounds. Recently, increasing attention has been paid to the role of miRNAs in the transmission of light signaling [3].

Light influences the transcription of *MIR* genes encoding miRNA, miRNA biogenesis, and RNA-inducible gene silencing complex (RISC) activity, thereby controlling not only the accumulation of miRNAs but also their biological functions. Since light regulates the processing of miRNAs, it is logical to assume that photoreceptors such as phytochromes, cryptochromes, and phototropins may be involved in the light-dependent changes in the level and content of miRNAs. This regulation is achieved due to the presence of light-sensitive elements in the microRNA promoters and can be caused by specific properties of light (red, blue, far-red, UVA, and UVB). *MIR* genes contain *cis*-elements in their promoter regions that bind certain light-dependent TFs such as PIF4 [4]. PIF4 interaction with the photoactivated form of phytochrome B is known to regulate a subset of downstream TFs by binding to the promoters of these genes [5,6]. The TF PIF4 functions at both transcriptional and posttranscriptional levels, regulating the biogenesis of miRNAs and binding directly to the promoters of the *MIR* gene [7]. Many families of light-dependent miRNAs are known: miR156/157, miR160, miR164, miR167, miR170/171, miR172, miR396, miR398, miR403 miR408, miR530, miR827, miR858, miR2876, and miR165/166 [8,9].

High irradiation levels, acting through the TF PIF4, control the expression of miR165/166. Similarly to high light intensity, low light intensity also alters the expression of miRNAs in plants. Shading at the reproductive stage increased the expression of several miRNAs associated with the cell wall, membrane, cytoskeleton, and cellulose synthesis and decreased the transcription of those involved in the control of photosynthesis, carbon and sugar metabolism, energy metabolism, and amino acid and protein metabolism. The 16 identified miRNAs and their 21 targets may improve shade tolerance and reduce crop losses [10].

It has been shown that small RNAs represent an independent mobile unit, and their mechanisms of movement between cells differ from the mechanisms of protein movement [11]. It was revealed that extracellular vesicles contained 10 families of miRNAs, which characterizes these lipid structures as a mechanism for the transport of small non-coding RNAs [12]. Vesicle transport has the potential to regulate the metabolic processes of target cells via gene silencing during RNA interference [13], as well as the response of plants to various environmental stress factors [14]. MiRNA miR165a is a mobile molecule that regulates the expression of the *PHABULOSA (PHB)* gene in plant leaves [15]. This gene is involved in various responses such as determining radial patterning in shoots [16], as well as vascular patterning [17], the promotion of thermotolerance [18], quiescent center-independent root meristem activities [19], and leaf polarity [20], many of which are photomorphogenic. The participation of miR165a in the regulation of other target genes, in particular, the *HD-ZIP III* genes: *ATHB-9/PHV*, *ATHB-14/PHB*, and *ATHB-15*, has been demonstrated [21]. These genes can be regulated by miR165a during the formation of the active form of phytochrome in the cell.

The interaction of miR165a with RISC reveals an identical mechanism in different organisms (such as the dicot Arabidopsis and the monocot maize) through the formation of an interfering complex with miR165a [15,21]. In Arabidopsis and rice, miR165/166 has been reported to mediate vascular development through binding its target genes [22,23]. The suppression of maize miR166 causes defective leaf polarity [24]. When miR165/166 accumulates in a cell, the expression of HD-ZIP III, PHB, PHV, REV, ATHB-8, or ATHB-15 factors is suppressed. This results in the formation of a regulatory network for transcription factors, leading to normal plant development. The activity of specific transcription factors leads to the correct expression of downstream genes responsible for tissue development [25].

The different roles of miRNAs in the organization of metabolic processes in plant cells have been demonstrated. Studies of the effects of UV light on *Chrysanthemum morifolium* plants have shown the participation of a number of miRNAs in the regulation of their target genes. In particular, three glycolysis-related genes, encoding glucan endo-1,3-β-D-glucosidase, glyceraldehyde-3-phosphate dehydrogenase, and pyruvate kinase, are targets of miR396f-5p, PC-5p-294053_21 and miR397a, respectively [26]. The potential for the interconnected regulation of carbon metabolism and secondary metabolism is revealed by the specialized transcription factors MYB28 and MYB29. These transcription factors not only regulate the glucose biosynthesis pathway but also influence several primary metabolic pathways for the synthesis of glucose precursors, including methionine biosynthesis, tryptophan biosynthesis, the tricarboxylic acid (TCA) cycle, sulfur metabolism, and the folate metabolism [4,6,27]. Recent studies have established a role for miR165/166 in auxin and abscisic acid (ABA) signaling, indicating a regulatory role for this microRNA in stress responses [28,29]. miR165/166 is involved in the regulation of the synthesis of cellular components and promotes resistance to stress, including through the control of carbohydrate metabolism [24]. The miR165/166-controlled regulation of HD-ZIP IIIs transcription factors mediates phytohormone signaling, which is essential in regulating leaf development and drought tolerance through metabolic coordination [30].

In the current study, we investigated the role of miR165a in the phytochrome signal transduction in plant leaves under changing light conditions. It is shown that the active form of phytochrome B in the cell causes a redistribution of miR165a between extracellular vesicles and leads to an increase in the level of the RISC-miR165a complex, which triggers the degradation of target mRNAs. This plays an essential role of light regulation of morphogenetic and metabolic processes.

## 2. Results

### 2.1. Estimation of the Amount of miR165a and of the Expression of the Genes Encoding the Proteins Regulating the Level of Micrornas in Maize Leaves upon Irradiation with Light of Different Wavelengths

**Figure 1:**
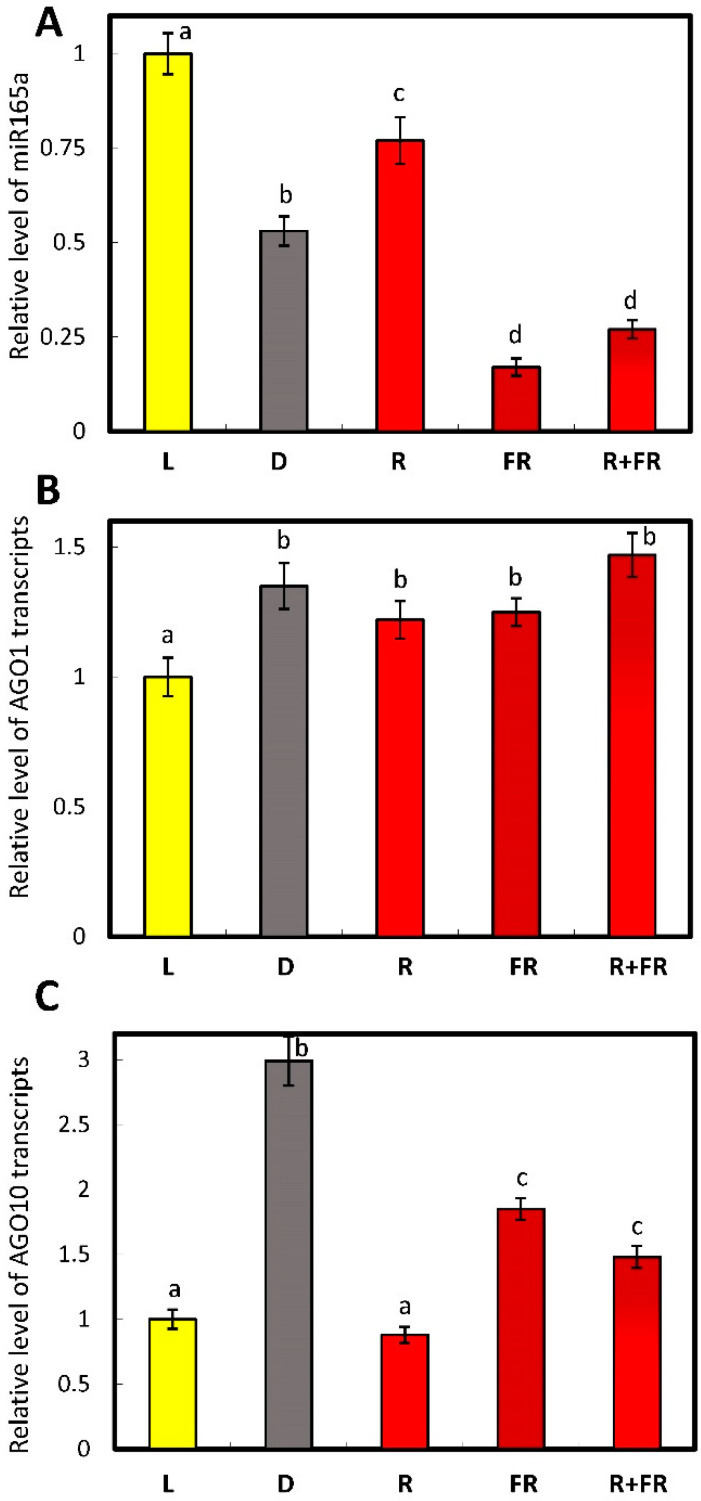
Relative levels of the transcripts of miR165a (**A**), AGO1 (**B**), and AGO10 (**C**) in maize leaves under different light conditions. Abbreviations: R, irradiation by red light; FR, irradiation by far-red light; and R + FR, irradiation by far-red light after red light. The data represent the means of three biological repeats ± SD (*p* ≤ 0.05), different letters indicate significant differences.

Quantitative real-time PCR was used to evaluate miR165a content in plant samples under different light conditions. This method allowed for the identification of the level of transcripts in samples under different light conditions. The results indicate that in the plants irradiated by white and red light, the content of miR165a was higher than in the plants incubated in darkness or irradiated by far-red light (Figure 1A). Consecutive irradiation of maize plants with red and far-red light causes a similar pattern of miR165a content to that of the far-red variant.

The level of transcripts of the genes encoding AGO1 and AGO10 proteins depends on the state of the phytochrome system in different ways. A study of the transcriptional activity of the *AGO1* gene showed very little observed effect or its absence upon the irradiation of the plants with the light of different wavelengths (Figure 1B). On the contrary, the transcripts of *AGO10* were highly accumulated in darkness, while red light strongly inhibited their accumulation, and far-red light reversed this effect only partially (Figure 1C). When the plants were irradiated by far-red light after red light, the levels of *AGO10* transcripts were similar to those observed upon irradiation by only far-red light (Figure 1C). Thus, the AGO10 levels and non-AGO1 levels were regulated by phytochrome.

### 2.2. Evaluation of miR165a Precursors in Maize Leaves upon Irradiation of Plants with Light of Different Wavelengths

**Figure 2:**
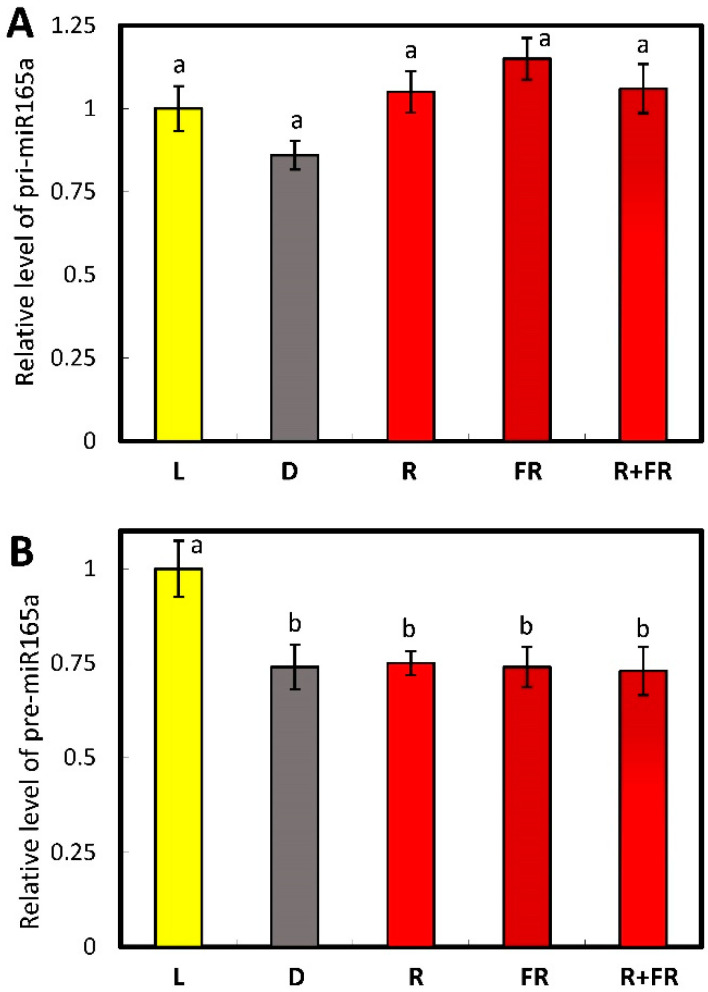
Contents of the transcripts of pri-miR165a (**A**) and pre-miR165a (**B**) in maize leaves under different light conditions. Abbreviations are the same as in Figure 1. The data represent the means of three biological repeats ± SD (*p* ≤ 0.05), different letters indicate significant differences.

The results on the influence of different light regimes on the level of pri-miR165a (initially transcribed long primary transcripts from the precursor gene of the corresponding miRNA) content indicate that there was no statistically significant change in pri-miR165a concentration in maize plants (Figure 2A). A study of the transcriptional activity of the miR165a gene, encoding the immediate stem-loop precursor pre-miR165a (the hairpin-shaped precursor processed into miRNA), showed a similar pattern to the pri-miR165a pattern in maize plants upon irradiation with light of different wavelengths (Figure 2B). Thus, both the pri-miR165a and pre-miR165a levels were not phytochrome dependent.

### 2.3. Determination of the Role of Phytochromes A and B in the Regulation of the Content of Free miR165a in Arabidopsis Leaves

We used Arabidopsis mutants for the genes encoding different types of phytochromes to identify the role of phytochrome A and B in the regulation of miR165a levels. Previously, we studied maize and Arabidopsis plants and revealed the uniformity of the response of gene expression in maize and Arabidopsis plants [31,32,33,34,35]. In accordance with these previous data, we assume that similar mechanisms exist for the miR165a role in light signal transduction in maize and Arabidopsis plants.

**Figure 3:**
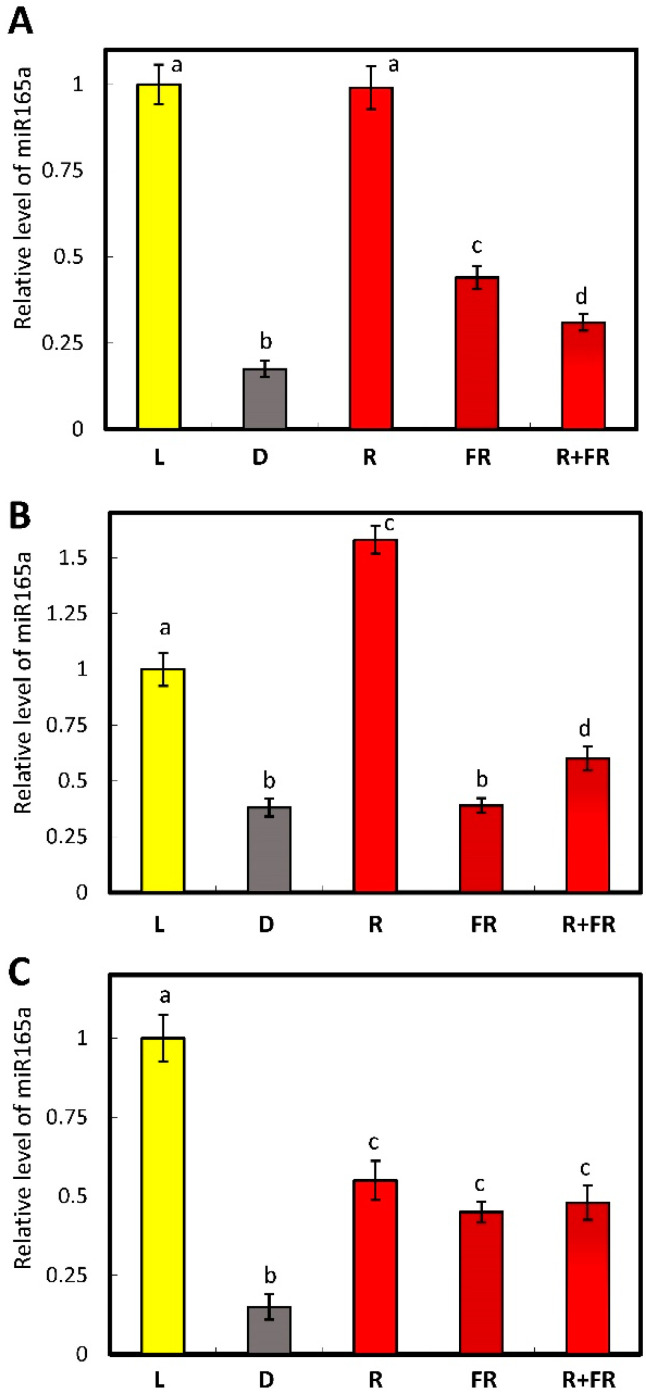
Relative levels of miR165 in leaves of *A. thaliana* plants under different light conditions: wild-type (**A**), phytochrome A knockout (**B**), and phytochrome B knockout (**C**). Abbreviations are the same as in Figure 1 and Figure 2. The data represent the means of three biological repeats ± SD (*p* ≤ 0.05), different letters indicate significant differences.

The irradiation of wild-type Arabidopsis plants with red light led to an increase in the content of mature miR165a as compared to dark-adapted plants, which equaled the level observed in the light-grown plants. On the contrary, the exposure of wild-type Arabidopsis plants to far-red light resulted in a decrease of the miR165a level, although it did not reach the level in darkness, and far-red light applied after red light did not reverse the effect (Figure 3A). In the mutants for the phytochrome A gene, the response of miR165a content to irradiation by red light was similar to what was observed in wild-type plants, and the level of miR165a was even markedly (by ~70%) higher than in the light-grown plants. The level of miR165a upon irradiation by far-red light was the same as in darkness, and the application of far-red light after red light was similar to the effect of far-red light (Figure 3B). In the mutants for the phytochrome B gene, exposure to the light of different wavelengths did not cause significant differences in the content of miR165a (Figure 3C).

### 2.4. Involvement of miR165a in Phytochrome-Dependent RNA Interference

**Figure 4:**
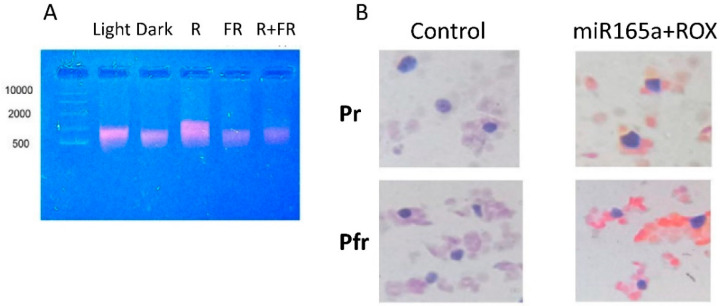
Phytochrome-dependent changes in the interfering complex with miR165a (**A**), and distribution of the interfering complex with miR165a (**B**), in maize leaf cells. Dark blue spots are nuclei; orange color corresponds to the fluorescent glow of the miR165a + ROX probe.

The use of the fluorescent probe miR165a-ROX for the analysis of total RNA from maize leaf cells under irradiation with light of different wavelengths made it possible to determine changes in the RNA interfering complex. It was shown that in the presence of an active form of phytochrome in the cell, a clearly detectable amount of the fluorescent probe bound to mRNA is observed, which indicates an intensification of RNA interference with the participation of miR165a (Figure 4A).

The staining of histological sections of maize plant leaves irradiated with the light of different wavelengths revealed the distribution of the RNA interfering complex with miR165a between different cell compartments. The amount of RNA interfering complex with miR165a increased significantly in the cytoplasm of maize leaf cells irradiated with red light (Figure 4B), indicating the involvement of miR165a in phytochrome-dependent RNA interference.

### 2.5. Involvement of miR165a in RNA-Dependent DNA Methylation

**Figure 5:**
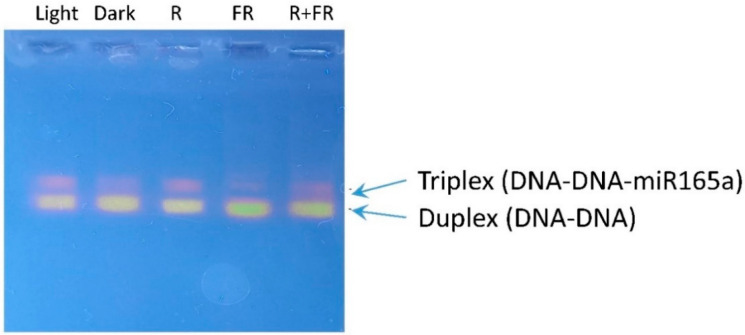
Assessment of the formation of the DNA-DNA-miR165a triplex structure in maize leaf cells upon irradiation of plants by light of different wavelengths.

Using a specific ROX probe for miR165a, the presence of triplex DNA in maize leaf cells, formed with the participation of the miRNA, was revealed, indicating the transcriptional mechanism of gene expression regulation through RNA-dependent DNA methylation with the participation of miR165a (Figure 5). The formation of the triplex DNA form was clearly dependent on the light regime being stimulated in the light, upon irradiation by red light, and being suppressed in darkness, and upon irradiation by far-red light.

### 2.6. Participation of Extracellular Vesicles in miR165a Transport

**Figure 6:**
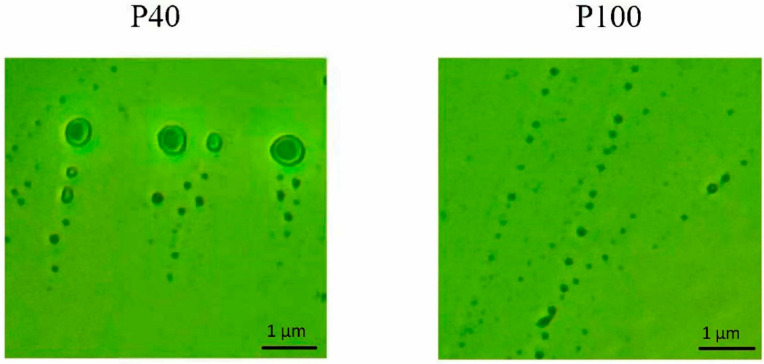
Typical images of extracellular vesicle fractions P40 and P100 taken using Olympus CX41 microscope with 1000× magnification. P40 is a fraction of vesicles obtained after differential centrifugation at a speed of 40,000 g, having a size of >0.170 µm. P100 is a fraction of vesicles isolated after centrifugation at a speed of 100,000 g and having a size of <0.147 µm.

The sequential high-speed centrifugation at 40,000 g and 100,000 g for 1 h made it possible to obtain two fractions of extracellular vesicles, P40 and P100. The microscopy of the P40 and P100 fractions made it possible to confirm the isolation of extracellular vesicles differing in their size (Figure 6). Fractions P40 and P100 were used to analyze the content of miRNA located inside and on the surface of extracellular vesicles.

**Figure 7:**
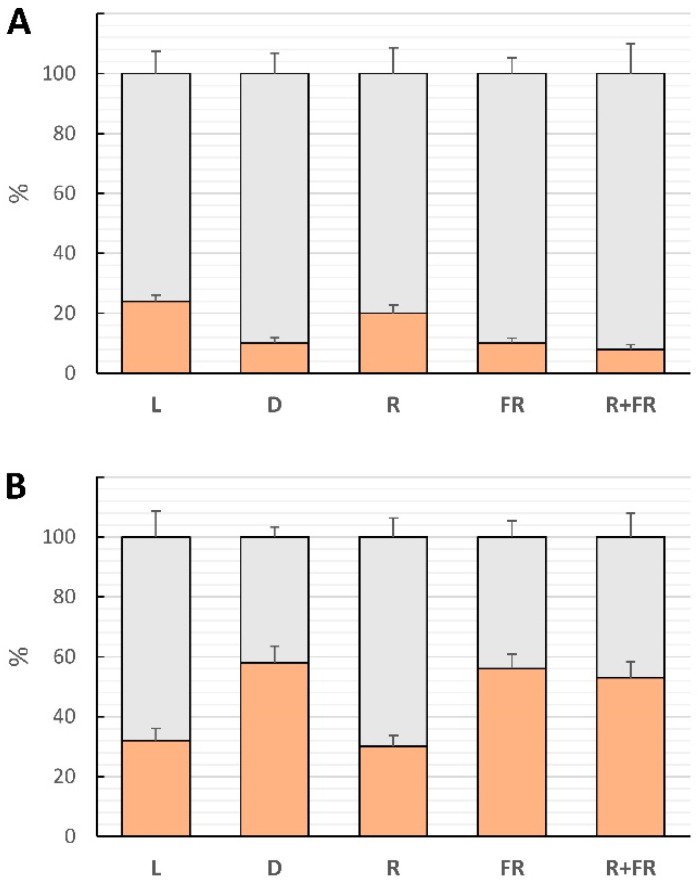
The content of miR165a in vesicles P40 (**A**) and P100 (**B**) of maize leaves under different light conditions. Light gray color—on the surface of vesicles; orange color—inside the vesicles. Abbreviations are the same as in Figure 1, Figure 2 and Figure 3. The data represent the means of three biological repeats ± SD (*p* ≤ 0.05).

After the treatment of vesicles of the P40 fraction with RNA nuclease and proteinase K, the content of miR165a revealed the changes on the surface of the vesicles and inside them (Figure 7A). In the vesicles isolated from light-incubated plants, the predominant location of miR165a was on the surface of the vesicles (~75%). The irradiation of maize plants with red and far-red light led to a change in the proportion of intravesicular miR165a fraction of P40. The distribution of miR165a between the surface and the interior of the vesicles was 79% and 21%, respectively, in the light and upon red light irradiation. Under these conditions, phytochrome is present in the cell in an active form. Irradiation by far-red light led to a decrease in miR165a inside the vesicles to <11%. A similar low level of miR165a was observed in darkness and after incubation by far-red light after red light. From the obtained data, we see that the active form of phytochrome reduces the proportion of miR165a located on the surface of vesicles.

To determine the location of miR165a on the surface or inside the vesicles of the P100 fraction, these samples were also treated with RNA nuclease and proteinase K. The obtained results of the quantitative assessment of the content of miR165a in the P100 fraction show the opposite pattern as compared to the P40 vesicles (Figure 7B). In the normal light conditions, the location of miR165a on the surface of the vesicles was almost 70%, and inside the vesicles, it was close to 30%. The presence of the active form of phytochrome (the experiment on irradiation by red light after darkness) resulted in the same distribution of miR165a between the surface and inner part of the vesicles as in the light-grown plants. In the dark-incubated plants, the pool of miR165a inside the vesicles increased to ~55%, and a lower amount (~45%) was on the surface of the P100 vesicles. The same distribution was observed in the plants irradiated by far-red light (Figure 7B). These data reveal the participation of extracellular vesicles in the miR165a transport, which is controlled by phytochrome.

## 3. Discussion

It was previously shown that light regime affects the activity of a number of enzymes through the regulation of the expression of their genes by changing the methyl status of their promoters [35,36,37,38]. An important mechanism for the light control of enzyme activity is the participation of plant cell photoreceptors, which specifically absorb the light quanta of certain wavelengths [39,40].

**Figure 8:**
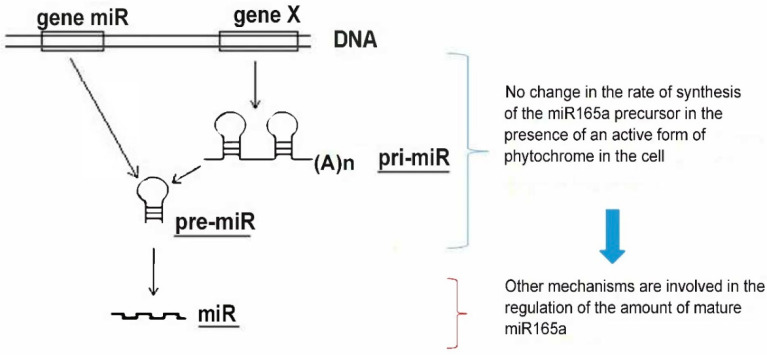
Changes in the content of mature miR165a in maize leaf cells upon irradiation with light of different wavelengths. Mechanism of formation of mature miR165a in maize leaves under irradiation with light of different wavelengths is not associated with a change in the intensity of synthesis of miR165a from its precursors. The absence of changes in the amount of pri- and pre-miRNAs when plants are irradiated with red and far-red light indicates that the transcriptional activity of the gene of this microRNA and the precursor gene do not change under these conditions. Fluctuations in the content of free miR165a in maize leaf cells may be associated with its participation in the process of RNA interference during the formation of the RISC-miR165a complex.

The results of this study show that the content of mature miR165a depends on the state of the phytochrome system. Moreover, it was found that the active form of phytochrome promotes the increase of the amount of analyzed miRNA. The levels of transcripts of the genes encoding AGO1 and AGO10 proteins depend on the state of the phytochrome system in different ways. The high content of *AGO10* transcripts in the dark is due to the dissociation of the phytochrome B factor-AGO10 complex, which contributes to an increase in the proportion of the bound form of miR165a in maize leaf cells (Figure 1C). An increase in AGO10 reduces the content of miR165a, which promotes the expression of target genes regulated by this miRNA. The scheme (Figure 8) illustrates this mechanism. Consequently, the results of the study on the phytochrome-dependent regulation of *AGO1* indicate the absence of an effect, which characterizes a constant level of post-transcriptional activity of miR165a in maize leaves (Figure 1B).

Changing the photoperiod conditions (light-dark transition) does not cause changes in the functioning of the genetic apparatus of the cell, which is reflected in the absence of statically significant changes in the amount of pri-miR165 in maize leaf cells (Figure 2A). The irradiation of maize plants with red and far-red light also does not lead to a change in the content of pri-miR165a relative to the dark variant, which indicates the absence of the phytochrome-dependent regulation of the synthesis of the analyzed miRNA. A Quantitative assessment of the content of pre-miR165a in maize plants in the presence of an active form of phytochrome in the cell (in the light and upon red light irradiation) showed no changes, which indicates a stable level of transcription of the miR165A gene (Figure 2B). A comparison of the results of studying the quantitative indicators of pre-miR165a and pri-miR165a in maize in the presence of active phytochrome in the cell indicates the absence of a phytochrome-dependent effect on the rate of synthesis of this miRNA.

The lack of complete restoration of the miR165a level to values in the dark upon the activation of phytochromes by red light is due to the peculiarities of the photoconversion of various types of phytochromes [41,42]. Phytochrome A (PhyA) does not have photoconversion, and the reverse transition to the inactive form occurs spontaneously after some time. However, phytochrome B (PhyB) is photoconvertible, so the lack of recovery to red light levels is due to its conversion to an inactive form upon exposure to light at a wavelength of 730 nm [43]. Sequential irradiation with red + far-red light leads to an increase in miR165a in maize leaves, which is associated with photoconversion of phytochrome B rather than phytochrome A. Similar results were obtained in Arabidopsis mutants, where the main role of PhyB in the regulation of miR165a was established. Since the interaction of miR165a with RISC is well studied and has an identical mechanism in different organisms, we assume that the formation of an interfering complex with miR165a in maize and Arabidopsis has a similar pattern.

The visualization of miRNAs by in situ hybridization can be used to obtain information about where and to what extent these molecules are distributed throughout the cells of a particular tissue, revealing the cellular localization of miRNA-mediated gene regulation [44]. An increase in the amount of the RNA interfering complex with miR165a in the cytoplasm of cells in the presence of the active form of phytochrome indicates the important role of miR165a in the transduction of the phytochrome signal, which ensures the control of target mRNAs in the plant cell.

Recent studies using Arabidopsis have revealed the important role of miRNAs in regulating the cell genome by controlling the methyl status of target genes. One of the most studied miRNAs is miR165/166 (miR165a), which provides a wide range of regulatory mechanisms of epigenetic control of the genome. Studies of this small RNA have shown the role of miR165/166 in the repression of a number of genes during plant development [25]. miR165 and miR166 have almost identical base sequences except for the CU at base 17. The Arabidopsis genome contains seven copies of miR166 genes and two copies of miR165 genes [25]. In the maize genome, these microRNAs are represented by their own genes. The formation of different subtypes of miRNAs of the 165/166 family is due to the expression of their own genes or differences in their maturation from the precursor, which is reflected in differences in the nucleotide composition. Both miRNAs can interact with the same type of target genes. The difference in the nucleotide composition of miR165 and miR166 in the 6–8 nucleotide region of the 5′ end of the miRNA, known as the “core” sequence, is generally considered necessary for the formation of RISC and provides specificity of interaction with the target gene [45,46]. In this work, we carried out the research (the development of a probe for RT-PCR, specific primers for RT-PCR, and a fluorescent probe) based on the nucleotide composition of miR165a, and therefore the current study is based specifically on the investigation of miR165a.

It has been established that an environmental light signal causes a change in the activity of miR165/166, which acts through the transcription factor PIF4. Photomorphogenesis is partially regulated by phytochromes and antagonistic PIF transcription factors. PhyB senses red light and inhibits PIF4 activity through ubiquitin-mediated proteasomal degradation [5]. When plants are exposed to red light, an increase in the expression of the *PIF4* gene has been established as a mechanism for compensating the pool of the degraded factor with the participation of the active form of phytochrome B [5,47]. Changes in the activity of the transcription factor PIF4, which has a high affinity for AGO10, promote the disassembly of the AGO10-miR165a complex and the inclusion of miR165a in AGO1, with the subsequent repression of gene expression [48]. Since PIF4 controls the activity of AGO10, which forms complexes with miR165/166 and causes its degradation, miR165/166 may play an important role in organizing the phytochrome-dependent gene expression regulatory mechanism. PIF4 directly represses the transcription of the *AGO10* gene [49].

Consequently, our results indicate the phytochrome-dependent regulation of miR165a content in plant leaves when irradiated with the light of different wavelengths. The signaling factor in this mechanism is the PIF4 factor, which controls the amount of AGO10 in the cell. In Arabidopsis, AGO10 is an important regulator that specifically interacts with miR166/165. The association is determined by the distinct structure of the miR166/165 duplex. It is noteworthy that the ability of AGO10 to bind miRNA does not affect its catalytic activity, and AGO10 has a higher binding affinity to miR165a than AGO1, a major miRNA degradation factor [50]. While miR165/166 exerts its actions through AGO1, AGO10 preferentially binds miR165/166, isolating it from AGO1 and leading to miRNA degradation, promoting the expression of target genes [49].

**Figure 9:**
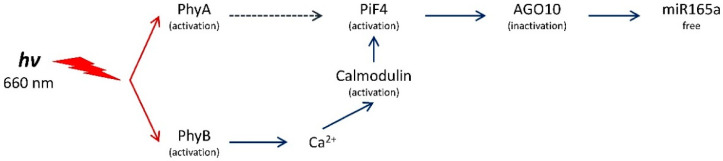
The mechanism of regulation of the light-dependent miR165a content via the phytochrome system.

The data obtained indicate that PhyB is mainly involved in the light regulation of miR165a levels, while PhyA may play an auxiliary role since Arabidopsis plants knocked out for the PhyB gene did not fully restore the miR165a level when irradiated with red light (Figure 3). The use of *A. thaliana* mutants for the phytochrome A and B genes demonstrated their role in the light control of the content of mature miR165a in maize leaf cells. The main photoreceptor that controls the amount of free miR165a in maize cells is phyB, while PhyA plays an auxiliary role (Figure 9).

Phytochrome B is not able to quickly penetrate the cell nucleus after photoexcitation [51], but it mediates the effect through the intracellular messenger Ca^2+^ [52,53]. This mechanism appears as the main pathway for regulating miR165a, which is reflected in a change in its amount in the cell after several hours, the time required to trigger the cascade mechanism of phytochrome B-dependent signal transduction. The specific Ca^2+^-dependent intranuclear mediators of this signaling pathway are calmodulins. They exhibit kinase activity, which promotes the degradation of PIF family factors, including PIF4 [54]. Changes in the activity of the transcription factor PIF4, which has a high affinity for AGO10, promote the disassembly of the AGO10-miR165a complex and the inclusion of miR165a in AGO1, causing the subsequent repression of gene expression [55]. Therefore, the phytochrome-dependent change in mature miR165a is associated with its release from the storage pool during its acceptance by AGO10 and not by a change in the rate of its synthesis from the precursor. In this scheme, phytochrome A plays an auxiliary role in the regulation of miR165a content in maize leaves. Since phytochrome A has the ability to quickly (within minutes) penetrate into the nucleus [56], in the first minutes after the irradiation of plants with red light, it can directly activate PIF4 through kinase activity. PIF4 also directly interacts with the bioactive form of PhyB through its APB motif. Although PIF4 does not have the APA motif required for interaction with PhyA, it still interacts with it at lower affinity than PhyB [5]. Upon irradiation with red light, PhyB is transformed into a biologically active form and interacts with PIF4, thereby initiating the phosphorylation, ubiquitination, and degradation of the latter [47,57,58]. A decrease in PIF4 in a cell reduces the transcriptional activity of the *AGO10* gene [59], which is reflected in an increase in free miR165a in cells and its acceptance by AGO1 for the further regulation of target genes at the posttranscriptional level [49].

The proportion of miR165a bound to genomic DNA is phytochrome-dependent since the amount of bound ROX probe changes depending on the light condition. The content of the miR165a-ROX-DNA complex depends on the state of phytochrome in maize leaf cells. The active form of phytochrome causes an increase in the number of triplex structures in the nucleus of plant cells (light-grown and red light irradiated plants). The amount of miRNA bound to the target DNA is regulated by PhyB since this type of phytochrome regulates the content of mature miR165a in maize leaf cells (Figure 3). Consequently, a PhyB-dependent increase in the content of mature miR165a in maize leaf cells when irradiated with red light causes the formation of the RNA-induced initiation of transcriptional gene silencing (RITS) complexes that carry out the formation of triplex elements with genomic DNA at the target site of this miRNA. RITS differs from RISC due to the presence of the chromodomain protein (Chp1) Argonaute 1 (Ago1) and a protein of the unknown function Tas3. It exhibits activity on gene expression through histone methylation [60].

MicroRNAs can act as epigenetic modulators by affecting key enzymes responsible for epigenetic responses, such as DNA methyltransferases (DNMTs), histone deacetylases (HDACs), and histone methyltransferases (EZHs) [61]. The genes containing sequences that promote triplex formation with miRNAs form triplex structures, suggesting a potential mechanism by which miRNAs can directly enhance gene expression. Adenine (A) and cytosine (C) content are determinants of miRNA binding to double-stranded DNA [62]. The formation of a DNA-DNA-miR triplex structure promotes targeted de novo DNA methylation through RNA-directed DNA methylation (RdDM) [63].

It has been shown that miR165/166 acts as a mobile signal involved in cell regulation, e.g., the expression of MIR165 and MIR166 is strongly detected in the abaxial epidermis of the leaf, but the amount of mature miR165/166 gradually decreases towards the adaxial side of the leaf. This gradient of miR165/166 in the leaf is formed through its intercellular movement, which mediates the regulation of the expression of *PHABULOSA (PHB)*, which encodes a class III homeodomain-leucine zipper transcription factor that determines leaf polarity [15]. Recent studies have shown that extracellular vesicles can carry miRNAs, serving the function of exchanging bioactive molecules and intercellular communication [64,65]. Plant extracellular vesicles are responsible for the transport of miRNAs, along with several RNA-binding proteins that promote selective loading and the stabilization of RNA into the vesicles [66].

Differential centrifugation is the most commonly used method for isolating extracellular vesicles from biological objects, including plants [67]. Moreover, the similar distribution of miR165a on the surface and inside the vesicles when maize plants are irradiated with far-red light and red plus far-red light indicates the key role of the photoconvertible phytochrome B in this process [43]. The results of studying extracellular vesicles of the P40 and P100 fractions made it possible to establish the redistribution of miR165a on their surface and inside them. The active form of phytochrome B causes a decrease in the proportion of surface-located miR165a in the P40 fraction, while the proportion of such miRNAs increases in the P100 fraction. The opposite picture is observed with a change in the content of miR165a inside the extracellular vesicles of the P40 and P100 fractions. The active form of phytochrome B increases the content of miR165a inside P40 vesicles and decreases it inside P100 vesicles.

## 4. Material and Methods

### 4.1. Object of Investigation

The leaves of 14-day-old maize (*Zea mays* L.) plants grown hydroponically under 12 h light and the leaves of 24-day-old *Arabidopsis thaliana* (L.) Heynh plants grown under 12 h light were used in this study. The seeds of the following lines of *A. thaliana* plants were used in this work: wild-type (Col-0, WT), the mutant for the phytochrome A apoprotein gene (*Phya-201*), and the mutant for the phytochrome B apoprotein gene (*Phyb-1*), obtained from the Max Planck Institute (Holm, Munich, Germany). During growth, the light intensity was 90 µmol quanta m^−2^ s^−1^. The experiment on irradiating plants with light of different wavelengths was carried out according to the previously described method [35].

### 4.2. RNA Isolation and Reverse Transcription

The isolation of total RNA from plant samples was performed by a modified phenol-chloroform extraction method using two precipitants: LiCl for high-molecular-weight RNA and PEG1500 for low-molecular-weight RNA [68].

Reverse transcription was performed using M-MuLV reverse transcriptase (SibEnzyme, Russia) according to the manufacturer’s instructions. To obtain the cDNA of total cell RNA, the primer Oligo(dT)15 Evrogen, Russia was used. The parameters for reverse transcription are as follows: incubation of the mixture at 70°C–5 min, 37°C–60 min, and 70°C–10 min.

To obtain the cDNA of the analyzed miRNA, a reverse transcription reaction was carried out with a specific probe developed for miR165a. The parameters for reverse transcription were as follows: incubation of the mixture at 16°C–30 min, 42°C–30 min, and 85°C–5 min [69]. When creating a specific stem-loop probe for the reverse transcription of the miRNA under study, a 44-nucleotide sequence of a stem-loop variant of HSV-1, 5′-GTCGTATCCAGTGCAGGGTCCGAGGTATTCGCACTGGATACGAC-3′ with the complement of six to the mature miRNA165a was used. The result of the formation of a specific probe for the quantitative assessment of mature miR165a using real-time PCR was a 70-nucleotide cDNA of the second strand of miR-HSV1, formed during reverse transcription.

### 4.3. Polymerase Chain Reaction

Polymerase chain reaction with gene-specific primers was performed using the AmpliSence reagent kit (Helikon, Russia). The reference gene was the elongation factor gene *ef-1ά* [70]. The development of primers for the quantification of miR-HSV1 was carried out on the basis of six additional nucleotides at the 3′ end of the miR-HSV1 sequence and the first 12 nucleotides of the 5′ end of the mature miR165a to form the forward primer sequence for real-time PCR. The reverse primer for real-time PCR was a segment of 16 nucleotides of the sequence at the 5′ end.

The nucleotide composition of miR165a primers was forward–5′-CACTGATCGGACCAGGCTTCA-3′; reverse–5′-GTCGTATCCAGTGCAGGGTCC-3′. The amplification parameters were as follows: preliminary denaturation–95 °C for 5 min, cycle–95 °C for 30 s, 58 °C for 30 s, 72 °C for 30 s (detection), and final elongation–72 °C for 10 min.

The content of pri-miR165a was assessed using a set of primers *mir165a*: forward–5′-CACTGATCGGACCAGGCTTCA-3′ and Oligo-dT_(15)_ primer [69]; pre-miR165a–using a set of primers: forward–5′-TCGGACCAGGCTTCATCCCC-3′ and reverse–5′-GGGGATGAAGCCTGGTCCGA-3′. The amplification parameters were as follows: preliminary denaturation–95 °C for 5 min, cycle–95 °C for 30 s, 53 °C and 58 °C for 30 s, 72 °C for 30 s (detection), and final elongation–72 °C for 10 min. Opticon MonitorTM Software (https://www.bio-rad.com/SearchResults?search_api_fulltext=opticon%20monitor%E2%84%A2%20software, accessed on 30 January 2024 Bio-Rad, Brea, CA, USA) was used to determine the relative levels of gene transcripts based on the 2^−ΔΔC^T method [71].

### 4.4. Analysis of RNA Interference and DNA Methylation

Analysis of RNA interference and RNA-dependent DNA methylation was carried out using the fluorescent probe ROX-miR165a, which is a nucleotide sequence complementary to the mature microRNA165a with the ROX fluorophore at the 3′ end. The results were assessed by electrophoresis in a 1% agarose gel. The intercalating dye for nucleic acid electropherograms was SybrGreen I. The photoexcitation of SYBR Green I was carried out by irradiation at 312 nm, and ROX emission was determined.

### 4.5. Subcellular Distribution of miRNA

An analysis of the subcellular distribution of miR165a was performed by in situ hybridization with paraffin-embedded plant leaf tissues [44]. The modified nucleotide ROX-miR165a, labeled at the 3′ end, was used as a probe.

The isolation of plant extracellular vesicles was carried out using the method of differential ultracentrifugation. From plant material (0.5 g), pure apoplastic liquid was collected by vacuum infiltration and centrifugation at 900× *g*. The isolation of two fractions of vesicles from the apoplastic fluid was carried out by successive stages of low-speed centrifugation at 2000× *g* and 10,000× *g* with further high-speed ultracentrifugation at 40,000× *g* and 100,000× *g* on a Beckman LB50 ultracentrifuge (Beckman, Brea, CA, USA) [72].

The visualization of extracellular vesicles after separation into fractions P40 and P100 was performed using the microscopy method on an Olympus CX41 microscope (Olympus, Tokyo, Japan) with a magnification of 1000×. The quantitative assessment of the content of nucleic acids in samples isolated from the P40 and P100 vesicle fractions and their purity was carried out spectrophotometrically on a NanoPhotometer C40 instrument (Implen, Munich, Germany).

To determine the position of miRNAs inside or on the surface of vesicles, fractions P40 and P100 were treated with 10 U proteinase K (SibEnzyme, Novosibirsk, Russia) for 15 min at 37 °C with Triton X-100. Then, the samples were treated with 10 U RNase (SibEnzyme, Russia) for 15 min at 37 °C. Enzyme inhibition after treatment was carried out by heating the reaction mixture at 70 °C for 5 min [72]. Immediately after proteinase and RNase treatment, total RNA was extracted by phenol-chloroform extraction for the further detection of specific miR165a.

### 4.6. Statistical Analysis

All experiments were performed in three biological and three analytical replicates. Data were subjected to two-way analysis of variance (ANOVA) using STATISTICA data analysis software version 9 (Statsoft Wipro, East Brunswick, NJ, USA). The results are presented as average means and standard deviations (SD). Statistically significant differences at *p* < 0.05 are discussed. Electrophoregram and microscopy images represent the data from typical experiments repeated three to four times.

## 5. Conclusions

Earlier studies demonstrated that miR165a is involved in the light-dependent mechanisms of the regulation of target genes [73]. For the quantification of miR165a, a specific probe was developed to quantify the mature 44-nucleotide stem-loop miRNA. The 70-nucleotide cDNA of the second strand of miR-HV1, formed during reverse transcription, makes it possible to quantify free miR165a using real-time PCR. The results of the study indicate that the content of miR165a depends on the state of the phytochrome system in the cell. Moreover, the active form of phytochrome promotes an increase in the content of this miRNA. The involvement of phytochrome B is demonstrated in the regulation of the level of miR165a in maize cells when the light regime of plants changes. An assessment of the amount of the miR165a precursor allowed us to conclude that the change in the content of free miR165a is associated with its binding with AGO10 and not with a change in the rate of its synthesis from the precursor.

**Figure 10:**
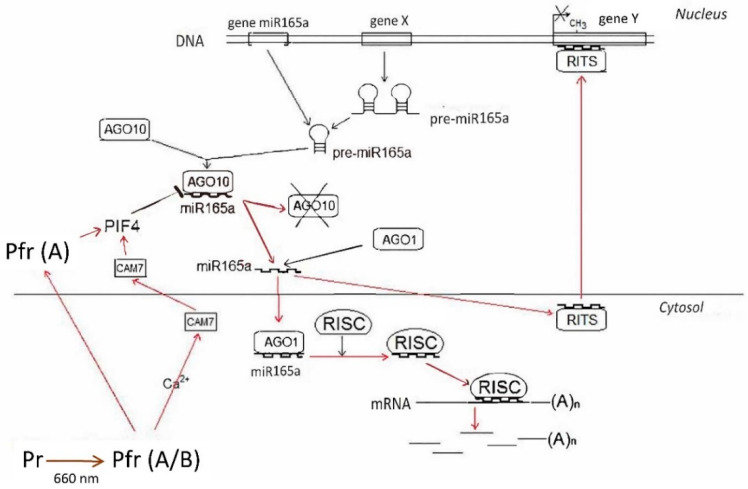
Participation of miR165a in phytochrome signal transduction in maize leaves upon changes in light conditions.

The use of knockout Arabidopsis plants for the phytochrome A and B genes made it possible to establish the physiological role of each of them in the transduction of the light signal in the plant cell. Phytochrome B plays a major role in the regulation of the content of free miR165a, but phytochrome A may play an auxiliary role (Figure 10). An increase in the level of miR165a-interfering complex in the cytoplasm of a maize leaf cell occurs during the formation of the active form of phytochrome after the irradiation of plants with red light. Consequently, miR165a in the cytoplasm of cells in the presence of the active phytochrome B carries out the transduction of a signal that controls the content of target mRNAs. It occurs via the formation of RITS complexes that promote the formation of triplex elements with genomic DNA at the target site of this miRNA. The formation of an active form of phytochrome leads to a redistribution of the amount of miR165a on the surface and inside the larger (P40) and smaller (P100) vesicles. The proportion of miR165a inside vesicles increases in the P40 fraction and decreases in the P100 fraction in the presence of an active form of phytochrome.

## Data Availability

The datasets generated for this study are available upon request from the corresponding author.

## Abbreviations

AGO1	ARGONAUTE1
AGO10	ARGONAUTE10
miRNA	microRNA
PhyA	phytochrome A
PhyB	phytochrome B
PIF	phytochrome-interacting factor
pri-miR	initially transcribed long primary transcripts from the precursor gene of the corresponding mi-croRNA
pre-miR	hairpin-shaped precursor processed into microRNA
RdDM	RNA-directed DNA methylation
TF	transcription factor
TCA cycle	tricarboxylic acid cycle
